# Soldiers’ load carriage performance in high mountains: a physiological study

**DOI:** 10.1186/s40779-017-0113-x

**Published:** 2017-02-17

**Authors:** Tirthankar Chatterjee, Debojyoti Bhattacharyya, Anilendu Pramanik, Madhusudan Pal, Deepti Majumdar, Dhurjati Majumdar

**Affiliations:** 1 Defence Institute of Physiology and Allied Sciences, Defence Research & Development Organisation, Ministry of Defence, Government of India, Lucknow Road, Delhi, 110054 India; 2 Retired from- Institute of Nuclear Medicine andAllied Sciences, Defence Research and Development Organization, Ministry of Defence, Government ofIndia, Delhi, India

**Keywords:** Load carriage, High altitude, Walking speed, Physiological responses

## Abstract

**Background:**

The present study was designed to evaluate load carriage performance at extremely high altitudes with different loads and walking speeds in terms of physiological evaluation. The degree of maximum oxygen consumption changes at high altitudes was also examined.

**Methods:**

Twelve Indian Army soldiers were acclimatized at altitudes of 3,505 m and 4,300 m. They walked for 10 minutes on a motorized treadmill at 2.5 km/h and 3.5 km/h speeds during carrying no loads and three magnitudes of load (10.7 kg, 21.4kg, 30 kg) at both altitudes. Physiological parameters such as oxygen consumption, energy expenditure, heart rate, and ventilation were recorded for each breath using a gas analyzer. The rating of perceived exertion was also noted after each load carriage session. Maximal oxygen consumption (VO_2max_) was measured at sea level and the two high altitudes, and respective relative workloads (% of VO_2max_) were calculated from oxygen consumption. Repeated measure ANOVA was applied to reveal the significant effects of the independent variables.

**Results:**

The participants had significant reductions in VO_2max_ with rising altitude. Marked increases in almost all physiological parameters were observed with increasing load, altitude, and speed. The soldiers expressed heavy perceived exertion levels with higher loads at 3.5 km/h at the two high altitudes.

**Conclusions:**

Considering the physiological responses, expressions of perceived exertion and changes in relative work load at both of the high altitudes Indian soldiers are advised to walk slowly with adequate rest in between their schedules and to carry not more than 32% of their body weight.

## Background

High altitude (HA) places a unique stress on humans, making it a distinctive research environment for exploring the physiological limits of the body. Changes in the environmental oxygen level at HA cause alterations in the oxygen levels in the lungs, arterial blood and eventually active muscles and tissues that in turn lead to adjustments in the respiratory and oxygen transport system [1]. Barometric pressure decreases with altitude, and the lower partial pressure of oxygen makes it less available for respiration [2]. Mountaineers, hikers and soldiers face difficulty adjusting to this condition. As a result of the low partial pressure of oxygen, hemoglobin is poorly saturated [3]. The resulting tissue hypoxia not only restricts the movements of the climber but also induces serious physiological, medical, sensory and neuro-behavioral problems [4]. Along with these issues, lower temperatures and strong winds compound the physiological stress at HA. The oxygen requirement of an exercise remains fixed regardless of altitude [5]. The maximum exercise capacity of a person thus decreases at high altitude. Bielderman et al. [6] reported that VO_2max_ decreased by approximately 30% at a simulated altitude of 4,298 m (acute exposure) and at Pikes Peak (4,298 m, chronic exposure). Fulco et al. [7] reported that the magnitude of sub-maximal exercise impairment is proportional to both the elevation and exercise duration at a given altitude, and that sub-maximal exercise performance at HA can improve with continued exposure without an increase in maximum aerobic capacity.

**Fig. 1:**
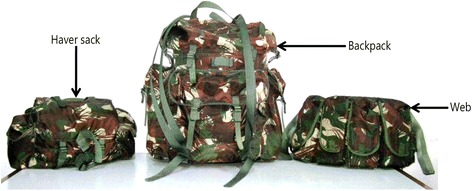
Different components of existing load carriage ensembles of Indian Army

Human-powered load carrying has always been a challenge in geographical areas where wheel-based transport is not available due to economical, technological or environmental limitations. Manual load carrying by Indian soldiers is often necessary in the remote countryside and mountainous areas, as these regions are mostly inaccessible to vehicles. In these regions, soldiers need to carry materials such as arms, ammunitions and rations for survival while ensuring maintenance of their work capacity and combat readiness. In the modern warfare scenario, the soldier must be properly loaded with maximum freedom of mobility. Indian infantry soldiers carry loads on the waist, back, shoulders and hands for marching orders [8]. They carry loads ranging from 10 to 30 kg over different terrain and in extreme environmental conditions. The composite load of existing load carriage ensembles (LCe) is 21.4 kg and consists of a backpack (BP, 10.7 kg), a haversack (HS, 4.4 kg), a web (2.1 kg) distributed on the back and waist and a hand-carried INSAS rifle (4.2 kg) [9] (Fig. 1). Pal et al. [10, 11] established standards of optimum load carriage at varying speeds and gradients for Indian Army soldiers on plains based on the energy cost, oxygen requirement and relative workload of the specific task. In the first study, they applied loads of up to 40 kg to Indian soldiers at varying speeds of 3.5 and 4.5 km/h. Loads of 36.1 and 21.4 kg were recommended for carriage at 3.5 and 4.5 km/h speeds. In the other study, loads of up to 21.4 kg were carried by Indian soldiers at a speed of 4.5 km/h at 0, 5, 10 and 15% gradients. A linear regression equation was applied to calculate the optimum load for each gradient and speed.

The Indian army deploys more than one hundred thousand soldiers in the high-altitude border areas. They are routinely subjected to load carriage operations in this difficult terrain. There are hardly any studies based on this field location which show the changes in physical performance capacity due to HA residency at different heights and the degree of physiological responses due to load carriage. It is strategically important to know this information for the deployment of soldiers to reduce the risk of morbidity and mortality among this population. It is hypothesized that the maximum aerobic capacity of soldiers will be significantly reduced with increasing altitude compared to sea level (SL). The amount of load to be carried by Indian soldiers will also be reduced with increasing altitude for a given walking speed. The present study was therefore designed to evaluate the performance of soldiers in terms of physiological responses while performing load carriage tasks at two walking speeds and two HAs with four magnitudes of load. The study also aimed to explore the degree of decrease in maximum aerobic capacity at HAs in comparison to SL. The establishment of guidelines for optimum load carriage for different altitudes was also planned based on the load carriage performance of the soldiers.

## Methods

### Participants

Twelve Indian Army soldiers with a mean (±SD) age of 26.8 (±3.9) years, height of 170.6 (±3.2) cm and weight of 66.2 (±6.8) kg participated in the study as volunteers. Physical characteristics of the soldiers with the changes in body weight at sea level and the two high altitudes are presented in Table 1. The participants were lowland natives of India. Eight of them were exposed to HA conditions for a second time, and the others were recent inductees. As per rules of the Indian Army, once a soldier completes his posting at HA, a gap of at least one year is provided before re-induction. Thus, the effect of acclimatization at HA of the previous posting is erased in this scenario. The volunteers were proscribed from smoking and alcohol consumption throughout the study period. They were allowed to perform jogging and light exercise in the morning and were relieved from night duty during the experiment.

**Table 1:** Physical characteristic of the volunteers

Parameter	Mean	SD
Age (year)	26.8	3.9
Height (cm)	170.6	3.2
Weight (kg)		
SL	66.2	6.8
HA1	66.3	7.2
HA2	65.7	6.9

### Ethical considerations

A clearance from the Ethical Committee of the author’s institute, which conforms with the recommendations of the Declaration of Helsinki (1983), was obtained before the study. After that, soldiers were briefed about the purpose and the risk of the experimentation and their informed consent was then obtained.

### Measurement of maximal aerobic capacity

The volunteers were first accustomed with a motorized treadmill (h/p/cosmos treadmill, Cortex Biophyzik Ltd, Leipzig, Germany) while walking on level ground at different speeds and gradients. On the day of the experiment, all participants reported to the laboratory at 0800 h after a light breakfast. During the measurement of maximal aerobic capacity, subjects wore a vest, underwear, shorts and physical training shoes. They were allowed to rest for one hour before the commencement of the experiment. After that, maximum oxygen consumption (VO_2max_) of the subjects was measured during a treadmill exercise with a regular increase in the gradient (Harbor protocol) [12] while keeping the speed constant. During the experiments, heart rate (HR), oxygen consumption (VO_2_), pulmonary ventilation (VE) and energy expenditure (EE) of each individual were recorded by gas analysis using a Meta-max 3B system (MMX 3B, Cortex, Leipzig, Germany). The corresponding HR at maximum VO_2_ was considered as the HR_max_ of the individual. This protocol was performed in Delhi (215 m, SL) and at two high altitude locations, Leh (3,505 m, HA1) and Tangtse (4,300 m, HA2), after full acclimatization.

### High altitude acclimatization

The soldiers of the present study were airlifted to Leh (HA1), and they moved from Leh to Tangtse (HA2) by road using an Army Vehicle. At high altitudes, subjects followed their routine acclimatization schedule as per existing Indian Army orders. At HA1, the acclimatization period was fixed for the initial 6 days as follows: 1st and 2nd days: Rest, except for short walks in unit lines only, not involving any climbs. 3rd and 4th days: Walk at slow pace of 1.5 to 3.0 km/h. Steep climbs were avoided. 5th and 6th days: Walk up to 5 km and climb up to 300 m at a slow pace. On the 7th and 8th days the volunteers were allowed to walk on the treadmill with a sub-maximal work load. The Lake Louis questionnaire was completed by each participant on the 3rd and 6th days of acclimatization. All experiments were conducted after the participants were declared medically fit for activity by a qualified medical doctor at HA1 and HA2. The participants rated approximately ‘2’ and ‘0’ on the Lake Louis questionnaire on the 3rd and 6th days of acclimatization, respectively. At HA2, the acclimatization period was 4 days as follows: 1st and 2nd days: Slow walk at a slow pace of 1.5 to 3.0 km/h, without any steep climbs. 3rd day: Slow walk and climb to 300 m. 4th day: Climb 300 m without equipment. On the 5th to 8th days, the subjects were permitted slow to moderate walks and treadmill exercises in the sub-maximal pattern. On the 9th day of stay at HA1 and HA2, VO_2max_ experiments were performed following the same protocol as SL with similar dress and experimental conditions to maintain the same number of exposure days at each altitude.

### Load carriage experiments

**Fig. 2:**
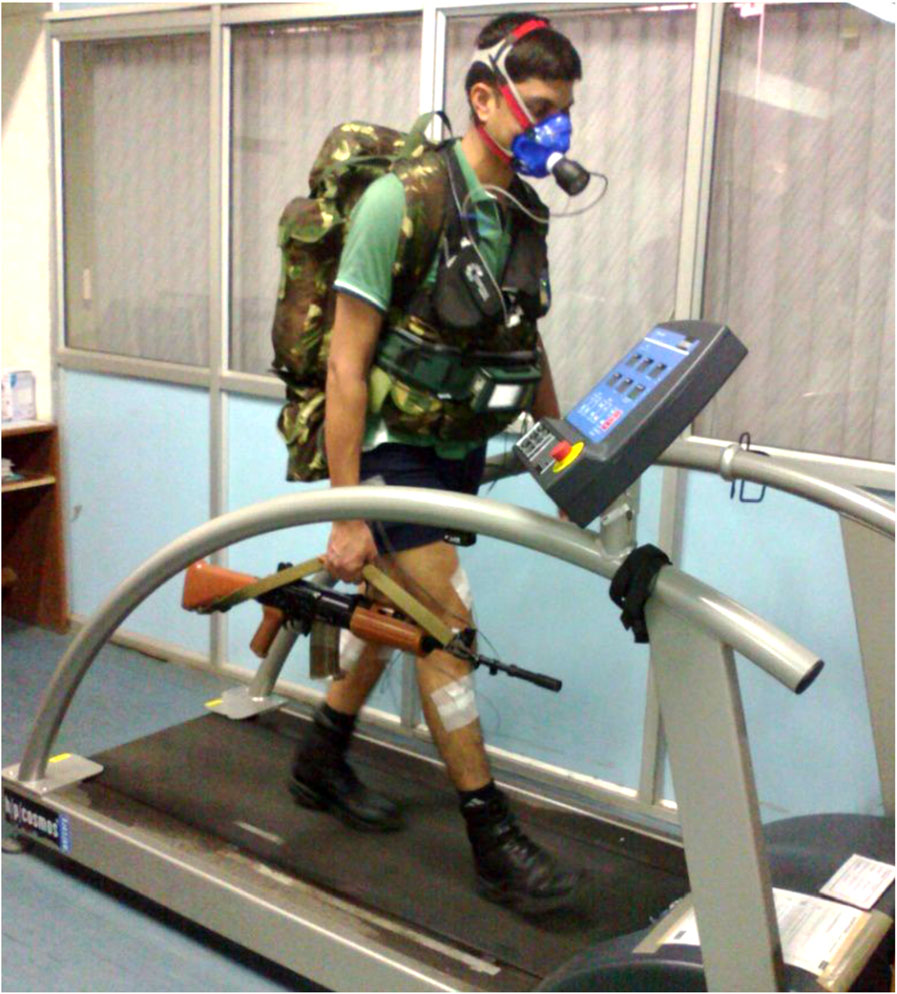
Soldier carrying 30 kg load at 3.5 km/h at HA1

The participants were subjected to treadmill walking for load carriage experiments at HA1 and HA2 on level ground without load (No load, NL) and with loads of 10.7 kg [L1- Haversack (HS), Web and Rifle], 21.4 kg [L2- Backpack (BP), HS, Web and Rifle] and 30 kg [L3- BP, HS, Web and Rifle] for 10 min in each experiment. The extra load of 30 kg was adjusted using the BP. Continuous treadmill walking at a fixed speed showed fluctuations in cardiorespiratory parameters for approximately 4–5 min to sense the effects of external physical factors such as load, gradient, speed and altitude, etc. After that, the cardiorespiratory system adapts to the external factors and shows a steady response. Hence, parameters derived from a 10 min exercise can be considered as reliable. The modes and magnitudes along with the percentages of body weight and placement of the loads are presented in Table 2. Each participant underwent a single load carriage trial per day to avoid any undue fatigue. The load carriage experiments were randomized to avoid any biases. The experiments were conducted in the temporary laboratory setup at both altitudes. Room air temperature and relative humidity (RH) were monitored by a digital thermometer-hygrometer. The volunteers wore T-shirts, shorts and boots during the load carriage experiments. The scientific equipment and other necessary stores/consumables used in the study were transported by road with Army trucks from Delhi to Leh and Leh to Tangtse and back. Walking speeds at the two altitudes were fixed at 2.5 and 3.5 km/h. The hypoxic conditions at high altitude, the associated difficulty levels of load carriage and suggestions given on optimum speed at given workloads by Nag et al. [13] were considered for the selection of walking speeds. Figure 2 shows a volunteer performing a load carrying task on a treadmill at HA1.

**Table 2:** Different components of load carriage ensembles with various load magnitides, its placement at body regions, and equivalence with percentage of body weight

Mode	Weight (kg)	Components	Percentage of body weight (%)
Existing load carriage ensemble of Indian Army	10.7	Haversack (HS)- at the waist, Web (WB)- at the front waist region, Rifle- hand-carried	16.2
Do	21.4	Back pack (BP)- back, HS, WB, Rifle	32.3
Do	30.0	BP, HS, WB, Rifle	45.3

### Parameters

Parameters such as VO_2_, HR, EE and VE were recorded by gas analysis of each breath using MMX 3B. The relative work load (RWL, %VO_2max_) was calculated as the percentage of VO_2max_ at respective altitudes using the formula VO_2_/VO_2max_ %. The MMX 3B system was thoroughly calibrated for volume, standard gas mixture, pressure and ambient air before commencement of experiments. Necessary information for ambient air pressure (barometric pressure, Pr) was obtained from the Indian air-force station at HA1 and the environment monitoring center at HA2. The participants performed load carriage tasks on a motorized treadmill for 10 min on each occasion. The same experimental protocol was followed at both altitudes. After each experiment, the participants rated their perceived exertion with a rating of perceived exertion (RPE) sheet (Borg’s 14-point scale) [14]. The average values of the last 3 min of the physiological parameters mentioned above were considered for statistical treatment.

### Statistical analysis

The statistical package for social sciences (SPSS, version 16.0, IBM Corporation, New York, USA) was used for statistical analysis. Repeated measure ANOVA was applied for the physiological and electromyographic parameters to determine significance across the conditions, as the same group of participants was exposed to two altitudes, two speeds and four loads in this study. The degree of freedom (df) of the data was checked through Mauchly’s test of sphericity for all independent variables and their combinations. If sphericity was not assumed, then it was corrected with the Greenhous-Geisser correction factor. The corresponding ‘F’ values were then taken as the level of significance. After determining the observed significance level for the various cardiorespiratory and electromyographic parameters, the Bonferroni post hoc test was applied for pair-wise comparison between the conditions. For all the tests, statistical significance was accepted at the *p* < 0.5 level. The same treatment was applied to the RPE data. One-way repeated measure ANOVA was used to check significance of VO_2max_ differences at the three altitudes.

## Results

Laboratory temperature, relative humidity and barometric pressure variations at sea level and the two HAs are presented in Table 3. The mean (± SD) VO_2max_ and HR_max_ at SL, HA1 and HA2 along with percent reductions are presented in Table 4. Variations in SpO_2_ at the two HAs are also included in Table 4. One way repeated measure ANOVA revealed that the changes are significantly different [*F*_(2, 22)_ =100.8, *P* < 0.5]. The Bonferroni test confirmed significant differences among the conditions when compared pair-wise. At HA1 and HA2, the average VO_2max_ of the volunteers were 83.6 and 70.8% of the capacity at sea level, respectively. The VO_2max_ at HA2 was 84.7% of that at HA1. The HR_max_ was reduced to 94.0, 93.2 and 99.1% when compared between SL and HA1, SL and HA2 and HA1 and HA2, respectively. One-way repeated measure ANOVA revealed that the changes were significantly different [*F*_(1.23, 13.52)_ =7.3, *P* < 0.5]. The Bonferroni post hoc test confirmed significant difference for pair-wise comparisons of SL and HA1 and SL and HA2.

**Table 3:** Changes in temperature, relative humidity, barometric pressure at SL and two high altitudes

Altitudes	Temperature (°C)	RH (%)	Pr (mmHg)
SL	27–30	33–40	720–726
HA1	22–28	18–35	507–516
HA2	16.5–21.5	35–60	436–447

*SL* sea level, *HA1* high altitude 1, *HA2* high altitude 2, *RH* relative humidity, *Pr* barometric pressure

**Table 4:** Changes in VO_2max_, HR_max_ and SpO_2_ at SL and high altitudes (mean ± SD, *n* = 12)

Parameter	SL	HA1	HA2	Percentage of decrease (%)		
				SL *vs* HA1	SL *vs* HA2	HA1 *vs* HA2
VO_2max_ (ml · min^−1^ · kg^−1^)	52.6 ± 3.8	44.0 ± 5.7	37.3 ± 4.0	16.4*	29.2*	15.3*
HR_max_ (beats · min^−1^)	187.6 ± 10.0	176.3 ± 14.3	174.8 ± 15.4	6.0*	6.8*	0.9
SpO_2_(%)		91.8	90.9			1.0

*P < 0.05. *SL* sea level, *HA1* high altitude 1, *HA2* high altitude 2

All physiological parameters increased with increasing load, speed and altitude. Increases in the physiological parameters are summarized in Table 5. Comparing HA2 to HA1, VO_2_ increased 17, 24, 15 and 16% under the four load conditions at 2.5 km/h. At 3.5 km/h, 22, 25, 26 and 12% increases were observed in the four loads from HA1 to HA2. The changes in VO_2_ were significant overall for altitude [*F*_(1, 11)_ = 211.1], speed [*F*_(1,11)_ = 196.5], load [*F*_(1.62, 17.85)_ = 57.1], and the interactions of altitude and speed [*F*_(1, 11)_ = 12.3] and altitude and load [*F*_(3, 33)_ = 5.6] (*P* < 0.5). Pair-wise comparisons revealed that VO_2_ remained significant in altitude, speed and load separately.

**Table 5:** Changes in physiological parameters and rating of perceived exertion under different loads, altitudes and speeds (mean ± SD, *n* = 12)

Parameters	2.5 km/h HA1				2.5 km/h HA2				3.5 km/h HA1				3.5 km/h HA2			
	NL	L1	L2	L3	NL	L1	L2	L3	NL	L1	L2	L3	NL	L1	L2	L3
VO_2_ (ml · min^−1^ · kg^−1^)*	9.3 ± 0.7	10.0 ± 0.9	11.4 ± 1.2	12.5 ± 1.3	10.3 ± 0.7	11.7 ± 0.9	12.5 ± 1.2	14.3 ± 1.3	11.0 ± 0.6	12.4 ± 1.3	13.3 ± 2.1	14.4 ± 2.2	12.5 ± 0.9	14.7 ± 0.7	15.8 ± 1.5	16.1 ± 1.8
HR (beats · min^−1^)*	87 ± 15	90 ± 12	92 ± 15	98 ± 12	89 ± 12	93 ± 12	98 ± 13	105 ± 11	89 ± 11	95 ± 14	100 ± 12	102 ± 10	96 ± 12	100 ± 10	109 ± 12	113 ± 16
VE (L · min^−1^)*	21.1 ± 3.0	23.7 ± 3.1	25.8 ± 2.5	28.8 ± 4.9	22.7 ± 3.8	26.8 ± 3.9	29 ± 4.5	32.2 ± 5.2	26.7 ± 3.2	29.9 ± 3.5	33.3 ± 4.9	35.2 ± 4.5	30.2 ± 4.6	35.7 ± 4.4	40.2 ± 4.6	41.8 ± 6.7
EE (Kcal · min^−1^)*	3.0 ± 0.3	3.2 ± 0.4	3.6 ± 0.3	4.0 ± 0.4	3.5 ± 0.4	4.0 ± 0.4	4.3 ± 0.4	4.6 ± 0.5	3.3 ± 0.4	3.8 ± 0.4	4.1 ± 0.6	4.6 ± 0.7	4.0 ± 0.4	4.7 ± 0.5	5.1 ± 0.6	5.2 ± 0.7
RWL (%)*	21.1 ± 2.5	22.6 ± 3.1	26.3 ± 3.9	28.7 ± 4.1	23.6 ± 4.0	26.8 ± 4.8	28.6 ± 4.8	32.9 ± 5.5	30.5 ± 2.8	34.6 ± 3.6	36.7 ± 4.4	39.3 ± 5.6	35 ± 3.6	40.4 ± 5.3	43.4 ± 5.2	44 ± 6.4
RPE*	9 ± 1.0	11 ± 1.4	12 ± 1.6	13 ± 1.2	9 ± 0.9	11 ± 1.0	13 ± 1.0	14 ± 0.9	8 ± 1.4	11 ± 1.4	14 ± 1.8	15 ± 1.7	10 ± 0.8	12 ± 1.2	15 ± 1.1	17 ± 1.2

*P < 0.05, over all significant. *NL* no load, *L1* 10.7 kg, *L2* 21.4 kg, *L3* 30 kg, *HA1* high altitude 1, *HA2* high altitude 2

Maximum HR (112.8 beat/min) was observed during carrying 30 kg at 3.5 km/h at HA2, which was 18% higher than that without a load. There were 2, 6, 9 and 4% increases in HR with zero, 10.7, 21.4 and 30 kg loads, respectively, at HA2 compared to HA1. On the other hand, HR increased 12, 8, 11 and 8%, respectively, under the four load conditions at 3.5 km/h at HA2 compared to HA1. The HR response was significant overall for altitude [*F*_(1, 11)_ =30.6], speed [*F*_(1, 11)_ =225.2], and load [*F*_(2.01, 22.12)_ =49.2]. The interactions of altitude and speed [*F*_(1, 11)_ =8.1] and speed and load [*F*_(3, 33)_ =4.6] (*P* < 0.5) were also significantly different. Pair-wise comparisons using the Bonferroni test revealed that HR remained significant in altitude, speed and load separately.

VE increased 27, 29, 26 and 22% under the four load conditions at 2.5 km/h at HA2 compared to HA1. At 3.5 km/h, 33, 34, 39 and 30% increases in VE were observed for the four loads from HA1 to HA2. VE escalated significantly (overall) for altitude [*F*_(1, 11)_ =233.1], speed [*F*_(1, 11)_ =157.6], load [*F*_(1.62, 17.83)_ =84.2], and the interactions of altitude and speed [*F*_(1, 11)_ =54.3], speed and load [*F*_(3, 33)_ =3.4] and altitude and load [*F*_(3, 33)_ =4.5] (*P* < 0.5). VE showed significant changes with altitude, speed, load, and the interaction between altitude and speed, altitude and load, and speed and load after pair-wise comparison using the Bonferroni test.

The effect of increasing HA was seen in case of EE, which increased 17, 23, 18 and 16%, respectively, at zero, 10.7, 21.4 and 30 kg loads at 2.5 km/h at HA2 compared to HA1. Similarly, at 3.5 km/h, 22, 25, 25 and 14% increases in EE were observed at the four loads from HA1 to HA2. The changes in EE were significantly (overall) high for altitude [*F*_(1, 11)_ =184.5], speed [*F*_(1, 11)_ =136.1], load [*F*_(1.31, 14.45)_ =76.1] and the interactions of altitude and speed [*F*_(1, 11)_ =9.7], altitude and load [*F*_(3, 33)_ =6.5] and speed and load [*F*_(3, 33)_ =84.4] (*P* < 0.5). Pair-wise comparisons revealed that EE remained significant in altitude, speed and load separately.

The RWL (%VO_2max_) for carrying a 30 kg load at 3.5 km/h speed at HA1 and HA2 were found to be 39.3 and 44.0%, respectively. There were 44, 50, 40 and 37% increases in RWL with zero, 10.7, 21.4 and 30 kg loads, respectively, at HA2 compared to HA1 at 2.5 km/h. On the other hand, RWL increased 49, 51, 52 and 34% under the four load conditions at 3.5 km/h at HA2 compared to HA1. In comparison, the changes in RWL for carrying the same loads at 2.5 km/h at both the altitudes were smaller. Overall significant increases in RWL were found for altitude [*F*_(1, 11)_ =211.1], speed [*F*_(1, 11)_ =196.5], load [*F*_(1.62, 17.87)_ =57.1] and the interactions of altitude and speed [*F*_(1, 11)_ =12.3] and altitude and load [*F*_(3, 33)_ =2.42] (*P* < 0.5). RWL was found to be significant in altitude, speed and load separately and the interactions between altitude and speed and altitude and load after post hoc analysis.

RPE was found to be significant with altitude, speed and load separately after the Bonferroni test. The RPE response was higher while carrying 30 kg loads at each altitude and speed. It showed a maximum response of 17, i.e., ‘very hard’, at HA2 at the 3.5 km/h walking speed with 30 kg. RPE was found to be significantly (overall) high for altitude [*F*_(1, 11)_ =18.5], speed [*F*_(1, 11)_ =24.3], and load [*F*_(3, 33)_ =147.8]. The interaction of altitude and load [*F*_(3, 33)_ =9.3] (*P* < 0.5) was also significant.

## Discussion

The maximum aerobic capacity of the participants in this study was measured at two different high altitudes (HA1 and HA2) on the 9th day of their stay, when they were well acclimatized and had no symptoms of acute mountain sickness (AMS). They showed significant decreases in physical performance capacity in terms of maximal aerobic power compared to SL. The decrease in VO_2max_ was approximately 16.4% at HA1 and 29.2% at HA2 in comparison to SL. Saunders et al. [15] observed almost similar 15–20% drops in VO_2max_ for athletes at HA. Sutton et al. [16] explained the decrease in VO_2max_ at HA by linking the oxygen transport chain with hypoxia and hypocapnia. They suggested the inactivation of the glycolytic pathway at maximum work load as a reason underlying this observation. Nag et al. [13] moved further and stated that exhaustion of the mechanism of ATP re-synthesis, which is required in active muscles during exercise at HA, led to unusual muscle fatigue and early withdrawal of the participants from maximal exercise at HA (3,660 m). Whatever the cause may be that the significant decrease in VO_2max_ of the individuals at HA affects their physical performance to a large extent. The degree of incapability depends on the rise of altitude and availability of ambient oxygen. It is expected that this decrease in aerobic capacity of the individuals would affect their load carriage ability. However, at the time of maximum exercise, the maximum HR at each altitude remained at least 10–12 beats/min (i.e., 6–7%) lower than at sea level. Saltin et al. [17] found 20–25 beat/min reductions in HR_max_ at HA. They attributed this event to diminished cardiac output and stroke volume. The SpO_2_ of the participants stabilized at approximately 92% at HA1 and approximately 91% at HA2 at rest. This kind of response is an indicator of less complete oxygenation of blood within the lungs during acclimatization at HA.

In this study, the physiological parameters such as VO_2_, HR, EE, VE and RWL significantly increased with increasing load, speed, and altitude, which indicated the degree of strain of the load carriage activity. The participants’ RPE also followed the same trend. Load carriage at high altitude means working in an environment with reduced atmospheric pressure, i.e., hypoxia. Such an activity increases oxygen requirements at HA, which must be accounted for in the face of this decreased oxygen pressure and availability. The elevated HR of the soldiers could be due to a compensatory mechanism to reduce stroke volume at HA as a result of reduced venous return [18]. Cibella et al. [19] observed marked increases in VE, respiratory power, and respiratory frequency at HA coupled with reduced diaphragm pressure and power output of exercise at HA. The authors suggested that the extremely high VE demand, requiring excessive respiratory power, may lead to fatigue of the diaphragm. In this context, Sutton et al. [16] postulated that a rise in ventilation at HA is associated with an increase in diffusion from capillaries to tissue mitochondria to compensate for oxygen unavailability at the tissue level. This could be considered as an important adaptive mechanism for acute exposure to HA. The participants in the present study had approximately 40% increases in VE from HA1 to HA2 during load carriage tasks. The soldiers followed the general acclimatization schedules and stayed at each HA for about one month. Their hyperventilation response can be considered as a natural approach to coping with the stresses of each altitude.

In the present study, VO_2_ increased significantly with increases in altitude, speed and load, along with other parameters. Cymerman et al. [20] derived the energy cost of load carriage with a backpack while walking on a treadmill at different gradients at an altitude of approximately 4,298 m. Deficient oxygen delivery and inability of the participants to continue the steady state exercise at HA were identified as a reason underlying the significant increase of EE at HAs. Nag et al.[13], while studying on Nepalese porters at HA, found elevated VO_2_ while performing load carriage tasks. The load was attached to the body with a head strap by the porters in their study. The authors reported extreme muscular fatigue and longer recovery time of the white fibers as underlying the rise in VO_2_. They concluded that the inability to continue a significant amount of work at HA might be the natural safeguard mechanism against overexertion. The results of the present study indicate the physiological response during load carriage only on the plain surface of HA. Hence, the degree and intensity of responses may not be consistent with other studies which were conducted on different gradients or rocky/icy mountain slopes. All participants of the present study were on the way to their respective HA postings. Thus, due to the paucity of time, data of load carriage tasks of similar intensity at sea level could not be recorded.

In the present study, the soldiers rated their perceived exertion levels while carrying L3 at HA2 as ‘Very hard,’ i.e., 17. At HA1, the RPE reached up to ‘15,’ i.e., ‘Hard/Heavy,’ for the same magnitude of loads. On the other hand, corresponding HR and VO_2_ responses were not as intense or equivalent for the same load carriage maneuvers. Possibly local (musculoskeletal) factors played important roles along with the cardiopulmonary response during load carriage at HAs. This could otherwise overwhelm the overall perception of exertion while working in this region [21–23]. The contribution of RWL, HR and VO_2_ to the central component of the perception of exertion was postulated by Pandolf et al. [24] and Pandolf [25]. The higher rating of perceived exertion of the soldiers at both altitudes might help them in preventing overexertion and the probability of injuries while performing the given tasks. This, in turn, helps preserve functional capacity of the physiological systems for further activities.

In the present study, Indian Army soldiers carried loads of approximately 16, 32 and 45% of their body weight (BW) at 2.5 and 3.5 km/h walking speeds at two HAs. As the soldiers need to be combat fit at HA with much lower VO_2max_ than sea level, guidelines or norms for load carriage at HA in terms of physiological responses must be considered as a great necessity. To carry out a task comfortably for 8 h per day, Saha et al. [26] recommended an acceptable workload (AWL) of 35% of the VO_2max_ for Indian Industrial workers. They used treadmill walking as a mode of exercise inside a laboratory to determine the oxygen cost and to subsequently determine the RWL. The present study was conducted inside field laboratory setups using a treadmill as the mode of exercise. In contrast, work load classifications for extreme environmental conditions such as HA are not available in the literature, nor has anyone raised any question to date about the formulation of separate norms for HA load carriage. Hence, the recommendations of Saha et al. [26] have been followed for calculating an acceptable work load at HA. The results of the present study show that at HA2 the RWLs are 39.3% for carrying 45% of BW at 2.5 km/h and 40.4, 43.4 and 44.0% for carrying 16, 32 and 45% of BW, respectively, at 3.5 km/h. Considering these facts, it can be said that carriage of 45% of BW and above cannot be permitted at an altitude of 4,300 m for more than 8 h at a speed of 3.5 km/h. The same is applicable for carriage of 45% of BW at 3,500 m. Instead, these loads are suitable for carriage for 2 h. However, a load below 32% of BW can be carried for 8 h with adequate rest pauses at a slow walking speed (approximately 2.5–3.5 km/h) at both altitudes. These suggestions resemble the predictions of Nag et al. [13], who recommended a load of 25–30 kg at a speed of 3.0–3.5 km/h as ideal for porters and highlanders. All the suggestions of load carriage presented in this article are meant for properly acclimatized persons without any clinical problems. These recommendations are based on a load carriage study on level ground at different altitudes. Hence, for climbing steep mountain gradients and walking through snowy tracks, these data may not be applicable.

## Conclusion

The participants of the present study had significant reductions in VO_2max_ and HR_max_ with rising altitude. Physiological responses increased with rising load, altitude, and speed. At the height of 3,500 m during level walking (0% gradient), load carriage of 32% of BW is allowed for long durations, such as 8 h, with necessary rest, water, and food. Forty-five percent of BW is recommended at this height for 2 h. At 4,300 m during level walking, load carriage operations of 32 and 45% of BW at a speed of 3.5 km/h are recommended for 2 h. Forty-five percent of BW is permitted for carriage for 8 h at a slower speed. Lower loads (below 32% of BW) would be ideal for carriage for long durations at both altitudes. To sustain the physiological stress and continue the load carriage activity at HA regions, the soldiers must walk slowly at approximately 2.5–3.5 km/h on level ground. These suggestions are based on load carriage performance of the participants on level ground at two altitudes. Climbing steep mountain gradients for long durations and subsequent fluid stress, exhaustion, and probable injuries are excluded in this study, thus the results are not applicable for these factors.

## Abbreviations

AMS: Acute mountain sickness
AWL: Acceptable workload
BP: Backpack
BW: Body weight
EE: Energy expenditure
HA: High altitude
HR: Heart rate
HS: Haversack
LCe: Load carriage ensembles
Pr: Barometric pressure
RH: Relative humidity
RPE: Rating of perceived exertion
RWL: Relative work load
SL: Sea level
VE: Pulmonary ventilation
VO_2_: Oxygen consumption
VO_2max_: Maximum oxygen consumption
